# Response to immunosuppressive therapy in PLA_2_R- associated and non-PLA_2_R- associated idiopathic membranous nephropathy: a retrospective, multicenter cohort study

**DOI:** 10.1186/s12882-017-0636-0

**Published:** 2017-07-10

**Authors:** Jia Wang, Qionghong Xie, Zhuxing Sun, Ningxin Xu, Yan Li, Liang Wang, Shaojun Liu, Jun Xue, Chuan-Ming Hao

**Affiliations:** 1 Division of Nephrology, Huashan Hospital, Fudan University, Wulumuqi Rd. (middle), Shanghai, 200040 China; 2 Division of Nephrology, Wuxi People’s Hospital, Qingyang Rd., Wuxi, Jiangsu province China

**Keywords:** Calcineurin inhibitors, Cyclophosphamide, Idiopathic membranous nephropathy, M type phospholipase A_2_ receptor, Immunosuppressive therapy, Remission

## Abstract

**Background:**

According to renal M type phospholipase A_2_ receptor (PLA_2_R) immunohistochemistry, idiopathic membranous nephropathy (IMN) could be categorized into PLA_2_R-associated and non-PLA_2_R-associated IMN. We conducted a retrospective, multicenter cohort study with 91 patients to compare the effect of immunosuppressive therapy between PLA_2_R-associated and non-PLA_2_R-associated IMN patients.

**Methods:**

A total of 91 biopsy-proven IMN patients from Huashan hospital and People’s Hospital of Wuxi in past 5 years were collected into this study. IMN with positive PLA_2_R immunohistochemistry in kidney biopsies were designated as PLA_2_R-associated IMN. Seventy-eight of the 91 IMN patients was PLA_2_R-associated IMN and 13 were non-PLA_2_R-associated IMN. Forty-five patients were treated with prednisone plus cyclophosphamide (CTX), and 46 with prednisone plus calcineurin inhibitors (CNIs). The follow-up duration was 15 months.

**Results:**

The total remission rate (76.9% versus 44.9%, *p* = 0.032) and complete remission rate (30.8% versus 2.6%, *p* = 0.003) were both significantly higher in the non-PLA_2_R-associated group than in the PLA_2_R-associated group at the 3rd month visit point, and at the 6th month time point, the complete remission rate was still significantly higher in the non-PLA_2_R-associated group (46.2% versus 11.5%,*p* = 0.007). But similar remission rates were found after the 9th month. Relapses were observed in 8 patients in PLA_2_R-associated group and none in non-PLA_2_R-associated group, although there was no significant difference between these two groups.

**Conclusion:**

Compared with the PLA_2_R-associated IMN, the non-PLA_2_R-associated IMN responded quicker to the immunosuppressive therapy.

## Background

Idiopathic membranous nephropathy (IMN) is one of the most common causes of adult nephrotic syndrome and its incidence is increasing dramatically in China [1]. The clinical course of IMN is heterogeneous: about 30% of patients have spontaneous remission; 20–40% of patients progress to renal failure; and the remaining patients maintain their proteinuria with normal renal function [2–4]. Among the patients without spontaneous remission, 70–80% was reported to respond to cyclophosphamide (CTX) or calcineurin inhibitors (CNIs) and have a better outcome than those who failed to respond to immunosuppressive therapies [5–9].

In 2009, Beck et al. identified an antibody against M-type phospholipase A_2_ receptor (PLA_2_R) in about 70% of IMN patients [10]. Recently another autoantibody that recognizes thrombospondin type 1 domain containing 7A (THSD7A) has been identified and is responsible for about 3% of IMN [11]. Studies showed that serum anti-PLA_2_R autoantibody (PLA_2_R-Ab) is a potential biomarker that is associated with disease activity, response to treatment and relapse [12–14]. Our previous study as well as others has shown that most of the patients who have positive serum PLA_2_R-Ab also have detectable PLA_2_R expression in the kidney along capillary loops; while those who have a positive PLA_2_R in the kidney tissue do not necessarily have positive serum PLA_2_R-Ab [15]. These data may suggest that kidney PLA_2_R expression is a more reliable marker for PLA_2_R-associated IMN, since serum PLA_2_R-Ab may turn negative in situations such as automatic remission or following treatment [15–18]. Accordingly, renal PLA_2_R can be used to classify PLA_2_R-associated IMN or non-PLA_2_R-associated IMN. Although strong evidence supports that subepithelial immunocomplex formation is a common mechanism responsible for the pathogenesis of MN, different autoantibodies may reflect different disease initiation and/or may lead to different target-antigen-associated changes, which may contribute to the heterogeneity of IMN. In the present study, we used renal PLA_2_R to classify IMN into PLA_2_R-associated and non-PLA_2_R-associated IMN and examine whether there was any difference in response to immunosuppressive therapy between these two groups.

## Methods

### Patients

Patients with biopsy-proven IMN and treated with immune-suppressants for at least 6 months in Huashan Hospital and People’s Hospital of Wuxi were included into this retrospective, multicenter cohort study from January 2008 to June 2014. Secondary causes of membranous nephropathy, such as lupus, hepatitis or malignancy, were excluded. This study was approved by ethic committee of Huashan Hospital, Fudan University.

### Renal PLA_2_R staining

**Fig. 1:**
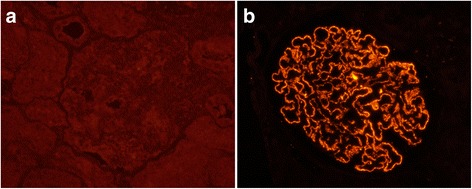
Renal PLA_2_R staining in IMN patients. (indirect immunofluorescence; original magnification ×400) **a** non-PLA_2_R-associated IMN; **b** PLA_2_R-associated IMN

Renal PLA_2_R of IMN patients was detected by indirect immunofluorescence in paraffin-embedded sections. Citrate buffer of pH 6.0 and microwaving at 100% power for 8 min were used for antigen retrieval and 3% bovine serum albumin was used for blocking. A commercial available anti-PLA_2_R antibody (produced in rabbit, Sigma, HPA012657) was diluted at 1: 500 and incubated at 4 °C over-night. The secondary antibody was a fluorescein Cy3-conjugated donkey anti-rabbit IgG antibody (Chemicon, AP182C) and diluted at 1: 200. Each case was run with a positive and negative control (secondary antibody only) (Fig. 1).

### Treatment protocols

The patients in CTX group received CTX intravenously with a dose of 0.5–0.75 g/m^2^(maximum of 1 g) monthly for six months and then once every 2 or 3 months. The initial dose of oral prednisone was 1 mg/kg/day (maximum of 70 mg/day) and tapered after 6–8 weeks. Ninety-six percent of the patients were treated with angiotensin converting enzyme inhibitors (ACEI)/angiotensin receptor blockers (ARB). The patients in CNIs group received an initial tacrolimus dose of 0.05–0.1 mg/kg/day or an initial cyclosporin A (CsA) dose of 3 mg/kg, which was then adjusted based on blood concentration. These patients visited the clinic every 2 weeks during the first 3 months of treatment. The blood trough concentration of tacrolimus was maintained in 5-10 ng/ml, and that of CsA was 100-150 ng/ml. The patients in CNIs group received oral prednisone at the dose of 0.5 mg/kg/day and tapered after 8–12 weeks. Ninety-four percent of the patients in CNI group were on ACEI/ARB.

### Definition of remission

Complete remission (CR) was defined as urinary protein excretion <0.3 g/24 h, with a normal serum albumin (ALB) concentration and a normal serum creatinine (SCR). Partial remission (PR) was defined as urinary protein excretion <3.5 g/24 h or a ≥ 50% reduction from peak value, accompanied by an improving or normalization serum ALB concentration and stable SCR. Total remission (TR) was defined as patients achieved CR or PR. Non-remission (NR) was defined when a patient received neither CR nor PR. Relapse in patients who had achieved CR or PR was defined as urinary protein excretion >3.5 g/24 h or >50% of the peak value, with a reduction of serum ALB concentration.

### Calculations and statistics

One-sample K-S testing was used to detect whether continuous variables were normal distribution. Normally distributed continuous variables were compared using the independent samples T test, and the results were expressed as mean values with standard deviations (mean ± SD). Abnormally distributed continuous variables were compared using two independent samples non-parametric test, and results were given as median (range interquartile). Categorical variables were compared using the χ2 test. A *p* value <0.05 was considered significant. The statistical analysis was performed using SPSS 13.0 software.

## Results

**Fig. 2:**
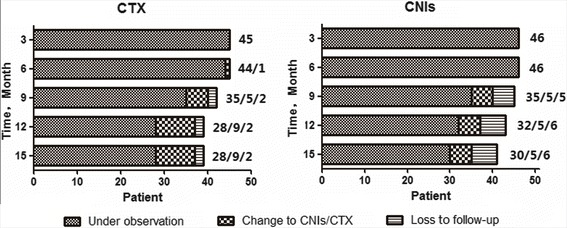
Available patients at every visit point

A total of 231 adult patients were diagnosed as IMN by kidney biopsy from January 2008 to June 2014 in Huashan Hospital and People’s Hospital of Wuxi. Among these patients, 189 were PLA_2_R-associated IMN and 42 were non-PLA_2_R-associated IMN. Ninety-one patients received immunosuppressive therapy for at least 6 months were included in the study by December 2014. Seventy-eight of them were PLA_2_R-associated IMN and 13 were non-PLA_2_R-associated IMN. Forty-five were treated with prednisone plus CTX, and 46 with prednisone plus CNIs. Follow-up was scheduled every 3 months. Nine patients in CTX group were switched to CNIs (8 NR patients, 1 PR patients who received transurethral resection of bladder neoplasm) and 2 patients lost to follow-up during the 15 months observation period. Six patients in CNIs group lost to follow-up and 5 NR patients were switched to CTX (Fig. 2).

**Fig. 3:**
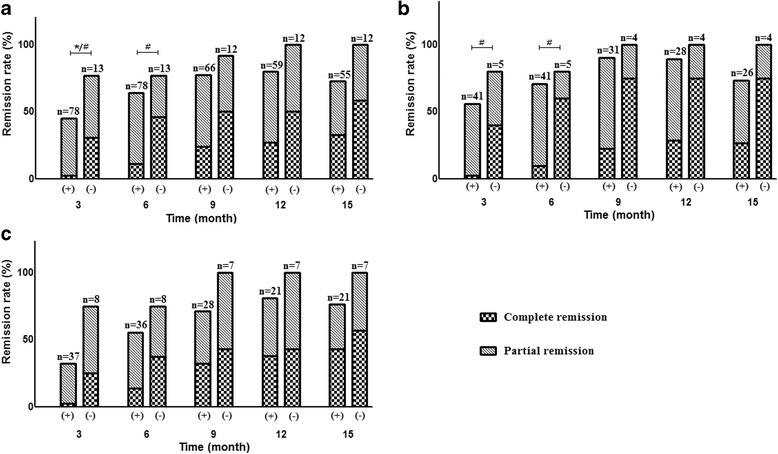
Remission rate of PLA_2_R-associated (+) and non-PLA_2_R-associated (−) group in 15-month observed period. **a** All patients treated with immunosuppressive therapy. **b** Patients treated with prednisone plus CNIs. **c** Patients treated with prednisone plus CTX. * There were significant differences with *p* < 0.05 in total remission rate between the two groups. # There were significant differences with *p* < 0.05 in complete remission rate between the two groups

Between the 78 PLA_2_R-associated IMN and 13 non-PLA_2_R-associated IMN patients, there were no significant differences in demographic or laboratory characteristic at baseline (Table 1). One third of the patients with PLA_2_R-associated IMN had previously been treated with ACEIs/ARB for 1.34 ± 2.39 months, whereas 38.5% of non-PLA_2_R-associated patients had been treated for 0.92 ± 1.26 months. No significant difference was observed in previous non-immunosuppressive treatments between the two groups. None of the patients in both groups received previous immunosuppressive treatment. The average time for TR and CR in non-PLA_2_R-associated IMN patients was 3.36 ± 1.91 and 5.50 ± 4.18 months, significantly shorter than TR (4.46 ± 2.39 months, *p* = 0.041) and CR (8.72 ± 3.31 months, *p* = 0.020) in PLA_2_R-associated patients. At the 3rd month point, both TR rate (76.9% versus 44.9%, *p* = 0.032) and CR rate (30.8% versus 2.6%,*p* = 0.003) in non-PLA_2_R-associated group were significantly higher than that in PLA_2_R-associated group. Although TR rate was similar in the two groups at the 6th month point, the CR rate in non-PLA_2_R-associated group was still higher (46.2% versus 11.5%,*p* = 0.007). All patients in non-PLA_2_R-associated group got remission at the 12th month point except one patient who lost to follow-up, and 79.7% of the patients in the PLA_2_R-associated group got remission. However, there was no significant difference between these 2 groups at this point (Fig. 3a). Relapse was found in 8/78 patients of PLA_2_R-associated group and 0/13 of non-PLA_2_R-associated group, but there was no statistical difference (*p* > 0.1). Similar results were found using intention-to-treat analysis at every follow-up point.

**Table 1:** Baseline characteristics between PLA_2_R-associated and non-PLA_2_R -associated groups

	PLA_2_R-associated	non-PLA_2_R-associated	*P* value
Patients	78	13	
CTX %	47.4% (37/78)	61.5% (8/13)	0.346
Gender (M:F)	52:26	5:8	0.102
Age (year)	53.81 ± 14.64	53.54 ± 17.52	0.953
Urine Protein (g/24 h)	5.51 (4.02, 7.72)	5.23 (2.34, 12.51)	0.829
Albumin (g/L)	19.45 ± 5.05	21.58 ± 9.07	0.423
Creatinine (μmmol/L)	78 (61.65, 93.75)	68.6 (60, 132.5)	0.875
Cholesterol (mmol/L)	7.86 ± 2.69	6.93 ± 1.88	0.238
Triglyceride (mmol/L)	2.1 (1.77, 3.21)	1.82 (1.57, 2.98)	0.434
Systolic Pressure (mmHg)	131.82 ± 17.99	121.54 ± 12.91	0.052
Diastolic pressure (mmHg)	80 (72, 90)	80 (66, 81)	0.180

**Fig. 4:**
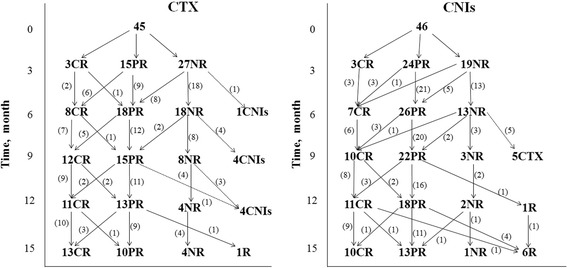
Remission and relapses in the CTX and CNIs group in 15-month observed period. (Abbreviations are: CR, complete remission; PR, partial remission; NR, non-remission; R, relapse)

Among the 91 patients in present study, 45 were treated with prednisone plus CTX, and 46 with prednisone plus CNIs. There were no significant differences in demographic or laboratory features at baseline between the two groups, except higher SCR in CTX group (*p* = 0.003) (Table 2). Thirty-eight percent of the patients in CTX group had previously been treated with ACEIs/ARB for 1.42 ± 2.51 months, whereas 30.4% in CNIs group for 1.14 ± 2.00 months. Other patients received immunosuppressive therapy immediately when they diagnosed with IMN. No significant difference was observed in previous non-immunosuppressive treatments between the two groups. Remission and relapse in CTX and CNIs group during the observation period were illustrated in Fig. 4. There was no significant difference in TR and CR between CTX and CNIs group during the 15 months of follow-up. One of the relapses in CNIs group occurred at the 12th month point, and 5 occurred at the 15th month point. The only relapse in CTX group occurred at the 15th month. The relapse rate was 4.2% in CTX group and 20.7% in CNIs group during the 15-month study period (*p* = 0.174). Side effects were observed in 7 patients in CTX group and 5 in CNIs group (*p* > 0.05, Table 2).

**Table 2:** Baseline characteristics between CTX and CNIs group

	CTX	CNIs	*P* value
Patients	45	46	
Gender (M:F)	28:17	29:17	0.935
Age (year)	55.18 ± 13.48	52.39 ± 16.34	0.377
PLA2R-associated %	82.2% (37/45)	89.1% (41/46)	0.346
Previously used ACEI/ARB %	37.8% (17/45)	30.4% (14/46)	0.460
Urine Protein (g/24 h)	5.87 (4.28, 9.53)	4.93 (3.66, 7.33)	0.096
Albumin (g/L)	19.48 ± 6.13	20.03 ± 5.45	0.650
Creatinine (μmmol/L)	94.95 ± 37.48	74.37 ± 23.93	0.003*
Cholesterol (mmol/L)	7.80 ± 2.30	7.64 ± 2.90	0.769
Triglyceride (mmol/L)	2.02 (1.78, 3.53)	2.09 (1.68, 3.04)	0.504
Systolic Pressure (mmHg)	132.05 ± 19.03	128.62 ± 16.24	0.364
Diastolic pressure (mmHg)	80.73 ± 9.33	80.82 ± 10.82	0.965
Side effects	7	5	0.509
Pneumonia	1	3	0.625
Hepatic dysfunction	2	1	0.985
Myelosuppression	1	0	0.495
Neoplasm of bladder	1	0	0.495
Intracranial hemorrhage	1	0	0.495
Gastrointestinal hemorrhage	1	0	0.495
Renal function deterioration	0	1	0.495

To remove the confounding of immunosuppressant, stratified analysis was processed according to CTX or CNIs therapy. Fifty-three percent (41/78, tacrolimus 32 and cyclosporine 9) of the patients in PLA_2_R-associated group and 38% (5/13, tacrolimus 3 and cyclosporine 2) in non-PLA_2_R-associated group were treated with CNIs, which was not significantly different between these 2 groups. In the patients treated with CNIs, CR rate in non-PLA_2_R-associated patients was higher than that in PLA_2_R-associated patients at the 3rd (*p* = 0.028) and 6th month point (*p* = 0.020), whereas no significant difference in TR rate between the two groups (Fig. 3b). In the patients treated with CTX, there was no statistical difference in both the TR and CR rate between non-PLA_2_R-associated and PLA_2_R-associated groups throughout the whole study period.

## Discussion

Although spontaneous remission occurred in about 30% of the untreated IMN patients, worsen renal function had been observed in another 20–40% patients, and they were recommended to receive immunosuppressive therapy [2–4]. Previous studies showed that the remission rates of prednisone plus CTX and prednisone plus CNIs were similar, so both of them were recommended as first line therapy for IMN patients. Since the finding of the autoantibody to podocyte antigen PLA_2_R in membranous nephropathy patients in 2009, accumulating evidences have shown that there was no significant difference in some relevant clinical parameters, such as age, gender, proteinuria or serum creatinine, between PLA_2_R-associated and non-PLA_2_R-associated IMN [16, 18], but there is a paucity of data regarding the treatment response between the PLA_2_R-associated and non-PLA_2_R-associated IMN. A recent study compared PLA_2_R-associated IMN patients who were serum PLA_2_R-Ab(−) with patients who were serum PLA_2_R-Ab(+), and found that patients who were serum PLA_2_R-Ab(+) exhibited higher levels of proteinuria and a lower chance of proteinuria remission [19]. However, serum PLA_2_R-Ab has been generally considered as a marker of disease severity and could be disappeared after immunosuppressive therapy or remission. Therefore, our study focused on the relationship between renal PLA_2_R and the treatment response to immunosuppressive therapy, and found that the TR and CR rates were both significantly higher in non-PLA_2_R-associated group than in PLA_2_R-associated group at the 3rd month visit point, and the CR rate was still significantly higher in the non-PLA_2_R-associated group at the 6th month time point. We also observed that relapses occurred in 8 out of 48 patients in PLA_2_R-associated group and none in non-PLA_2_R-associated group, although there was no significant difference.

The reason of faster response to immunosuppressive treatment in non-PLA_2_R-associated IMN patients than in PLA_2_R-associated IMN is unclear. Renal PLA_2_R has been identified as a major target antigen in about 80% IMN patients [15, 20]. In renal PLA_2_R negative IMN patients (non-PLA_2_R associated), alternative target antigens in podocyte other than PLA_2_R could be responsible for the disease, such as recently identified thrombospondin type-1 domain-containing 7A (THSD7A) in about 10% of non-PLA_2_R-associated IMN patients [11]; Cationic bovine serum albumin could cause childhood IMN, and children who had high levels of both circulating cationic BSA and BSA-specific antibodies were negative for anti-PLA_2_R antibodies [21]; Antibodies against the superoxide dismutase 2 (SOD2) was found in serum and glomeruli of IMN patients but not in patients with other glomerular diseases or normal kidney and might cause IMN [22, 23]. Different auto-antigen targeted by an antibody may give rise to a different response to treatment. We then analyzed the remission stratified by suppressants CTX or CNIs and also found that the patients treated with CNIs had higher CR rate in non-PLA_2_R-associated group than in PLA_2_R-associated group at the 3rd and 6th month. In patients treated with CTX, the total remission rate (75% versus 32.4%, *p* = 0.067) and complete remission rate (25% versus 2.7%, *p* = 0.077) tended to be higher in non-PLA_2_R-associated group at the 3rd point, but unfortunately the difference was not statistical significant. This may be attributed to the small sample size after stratification.

In the present study, the treatment protocol of CTX was pulsed intravenous CTX once every month for 6 months and then once every 2 or 3 months, plus oral prednisone daily. Intravenous CTX was used because oral CTX is not available in our area. The remission rate was 82.1% after 15 months of treatment, similar to the rate in another study from China (87.5% at the 12th month point, 92.9% at the 18th month point) [24], and consistent with other studies used Ponticelli or Dutch method. According to Ponticelli protocol, which is a 6-month regimen consisting of daily oral CTX alternating monthly with corticosteroids, the remission rates are 72–93% [5, 6]; In Dutch protocol, which used oral CTX daily for 12 months plus oral prednisone daily or every other day for 6 months, the remission rates are 84–93% [9, 25]. So the remission rate of intravenous CTX in the present is similar to oral CTX protocol in the literature, but total CTX accumulation in intravenous CTX protocol is much less compared to oral protocol. In the CNIs group, the remission rate was 76.7%, also consistent with the literature that shows the remission rate of 75–85% in the patients with CsA plus prednisone treatment [26, 27], and 79–87.5% with tacrolimus plus prednisone treatment [24, 28].

There are some studies compared CTX and CNIs in the treatment of IMN. Two trials from China both drew a conclusion that response was quicker in CNIs group compared with CTX group [24, 28]. Whereas, a study from India, which included non-immunosuppressive therapy resistant IMN patients, observed a similar remission rate at the end of 6 and 12 months between CTX and CNIs groups, and the difference with these studies may be explained by genetic differences and patient selection criteria [29]. In our study, TR rate was 58.7% in CNIs group whereas 40% in CTX group at the 3rd month visit point, but difference was not significant (*p* = 0.075). And there were no significant differences in TR and CR rate between the two groups after 6 months of follow-up. In previous studies, relapse was found in 13–50% patients treated by CNIs within 1 year of drug withdrawal [7, 28], but there was no significant difference when compared to the CTX plus corticosteroids treatment [24, 27, 28]. In our study, relapses tended to occur more often in the CNIs group but the difference was not significant (20.7% versus 4.2%, *p* = 0.174). If the sample enlarged, more difference between the two groups might be found.

There were several limitations in our study. Firstly, it’s a non-randomized retrospective study. However, in our study, clinical doctors were not informed whether a patient was PLA_2_R-associated or non-PLA_2_R-associated IMN when they decided which immunosuppressive drug would be used, and there was no significant differences in baseline characteristic between PLA_2_R-associated and non-PLA_2_R-associated group. Baseline serum creatinine of CTX group was higher than that of CNI group, because patients with high baseline serum creatinine were tend to be treated with CTX, considering the effect of CNIs on renal function. Secondly, in the present study, only 1/3 of patients received ACEI or ARB before immunosuppressive therapy, since the non-immunosuppressive therapy using ACEI/ARB for at least 6 months before immunosuppressive therapy was not a standard practice in China before KDIGO guideline was released, particularly for patients with heavy proteinuria. However, according to our experience, in patients with severe nephrotic syndrome, spontaneous remission will not usually occur until one year after supportive therapy, mostly one and half years later. This experience is consistent with the literature [2]. Thirdly, the offending antibody in non-PLA_2_R-associated IMN is not known, and could be heterogeneous. Finally, the sample size of the present study was small, particularly the number of patients with non-PLA_2_R-associated IMN. PLA_2_R-associated-IMN patients account for 85.7% in our study, higher than 74% in the literature [17], which also made the inclusion of non-PLA_2_R-associated IMN patients more difficult. Therefore, large sample size, prospective controlled and randomized trials are needed to draw a more definitive conclusion.

## Conclusion

Renal PLA_2_R could be an important factor to predict the treatment response of IMN. Compared with PLA_2_R-associated-IMN, patients with non-PLA_2_R-associated IMN responded faster to the immunosuppressive therapy.

## Abbreviations

ACEI: Angiotensin converting enzyme inhibitors
ALB: Albumin
ARB: Angiotensin receptor blockers
CNIs: Calcineurin inhibitors
CR: Complete remission
CsA: Cyclosporin A
CTX: Cyclophosphamide
IMN: Idiopathic membranous nephropathy
NR: Non-remission
PLA_2_R: Phospholipase A_2_ receptor
PLA_2_R-Ab: Anti-phospholipase A_2_ receptor autoantibody
PR: Partial remission
SCR: Serum creatinine
TR: Total remission

## References

[1] Pan X, Xu J, Ren H, Zhang W, Xu Y, Shen P, Li X, Wang W, Chen X, Wu P (2013). Changing spectrum of biopsy-proven primary glomerular diseases over the past 15 years: a single-center study in China. Contrib Nephrol.

[2] Polanco N, Gutierrez E, Covarsi A, Ariza F, Carreno A, Vigil A, Baltar J, Fernandez-Fresnedo G, Martin C, Pons S (2010). Spontaneous remission of nephrotic syndrome in idiopathic membranous nephropathy. J Am Soc Nephrol.

[3] Cattran D (2005). Management of membranous nephropathy: when and what for treatment. J Am Soc Nephrol.

[4] Schieppati A, Mosconi L, Perna A, Mecca G, Bertani T, Garattini S, Remuzzi G (1993). Prognosis of untreated patients with idiopathic membranous nephropathy. N Engl J Med.

[5] Ponticelli C, Altieri P, Scolari F, Passerini P, Roccatello D, Cesana B, Melis P, Valzorio B, Sasdelli M, Pasquali S (1998). A randomized study comparing methylprednisolone plus chlorambucil versus methylprednisolone plus cyclophosphamide in idiopathic membranous nephropathy. J Am Soc Nephrol.

[6] Jha V, Ganguli A, Saha TK, Kohli HS, Sud K, Gupta KL, Joshi K, Sakhuja V (2007). A randomized, controlled trial of steroids and cyclophosphamide in adults with nephrotic syndrome caused by idiopathic membranous nephropathy. J Am Soc Nephrol.

[7] Praga M, Barrio V, Juarez GF, Luno J (2007). Tacrolimus monotherapy in membranous nephropathy: a randomized controlled trial. Kidney Int.

[8] Fritsche L, Budde K, Farber L, Charisse G, Kunz R, Gaedeke J, Neumayer HH (1999). Treatment of membranous glomerulopathy with cyclosporin a: how much patience is required?. Nephrol Dial Transplant.

[9] Hofstra JM, Branten AJ, Wirtz JJ, Noordzij TC, du Buf-Vereijken PW, Wetzels JF (2010). Early versus late start of immunosuppressive therapy in idiopathic membranous nephropathy: a randomized controlled trial. Nephrol Dial Transplant.

[10] Beck LJ, Bonegio RG, Lambeau G, Beck DM, Powell DW, Cummins TD, Klein JB, Salant DJ (2009). M-type phospholipase A2 receptor as target antigen in idiopathic membranous nephropathy. N Engl J Med.

[11] Tomas NM, Beck LJ, Meyer-Schwesinger C, Seitz-Polski B, Ma H, Zahner G, Dolla G, Hoxha E, Helmchen U, Dabert-Gay AS (2014). Thrombospondin type-1 domain-containing 7A in idiopathic membranous nephropathy. N Engl J Med.

[12] Hofstra JM, Beck LJ, Beck DM, Wetzels JF, Salant DJ (2011). Anti-phospholipase a(2) receptor antibodies correlate with clinical status in idiopathic membranous nephropathy. Clin J Am Soc Nephrol.

[13] Beck LJ, Fervenza FC, Beck DM, Bonegio RG, Malik FA, Erickson SB, Cosio FG, Cattran DC, Salant DJ (2011). Rituximab-induced depletion of anti-PLA2R autoantibodies predicts response in membranous nephropathy. J Am Soc Nephrol.

[14] Hoxha E, Harendza S, Zahner G, Panzer U, Steinmetz O, Fechner K, Helmchen U, Stahl RA (2011). An immunofluorescence test for phospholipase-a(2)-receptor antibodies and its clinical usefulness in patients with membranous glomerulonephritis. Nephrol Dial Transplant.

[15] Xie Q, Li Y, Xue J, Xiong Z, Wang L, Sun Z, Ren Y, Zhu X, Hao CM (2015). Renal phospholipase A2 receptor in hepatitis B virus-associated membranous nephropathy. Am J Nephrol.

[16] Hoxha E, Kneissler U, Stege G, Zahner G, Thiele I, Panzer U, Harendza S, Helmchen UM, Stahl RA (2012). Enhanced expression of the M-type phospholipase A2 receptor in glomeruli correlates with serum receptor antibodies in primary membranous nephropathy. Kidney Int.

[17] Debiec H, Ronco P (2011). PLA2R autoantibodies and PLA2R glomerular deposits in membranous nephropathy. N Engl J Med.

[18] Svobodova B, Honsova E, Ronco P, Tesar V, Debiec H (2013). Kidney biopsy is a sensitive tool for retrospective diagnosis of PLA2R-related membranous nephropathy. Nephrol Dial Transplant.

[19] Qin HZ, Zhang MC, Le WB, Ren Q, Chen DC, Zeng CH, Liu L, Zuo K, Xu F, Liu ZH. Combined assessment of Phospholipase A2 receptor Autoantibodies and Glomerular deposits in membranous nephropathy. J Am Soc Nephrol. 2016; 10.1681/ASN.2015080953 PMC5042668 26989120

[20] Qin W, Beck LJ, Zeng C, Chen Z, Li S, Zuo K, Salant DJ, Liu Z (2011). Anti-phospholipase A2 receptor antibody in membranous nephropathy. J Am Soc Nephrol.

[21] Debiec H, Lefeu F, Kemper MJ, Niaudet P, Deschenes G, Remuzzi G, Ulinski T, Ronco P (2011). Early-childhood membranous nephropathy due to cationic bovine serum albumin. N Engl J Med.

[22] Prunotto M, Carnevali ML, Candiano G, Murtas C, Bruschi M, Corradini E, Trivelli A, Magnasco A, Petretto A, Santucci L (2010). Autoimmunity in membranous nephropathy targets aldose reductase and SOD2. J Am Soc Nephrol.

[23] Buelli S, Perico L, Galbusera M, Abbate M, Morigi M, Novelli R, Gagliardini E, Tentori C, Rottoli D, Sabadini E (2015). Mitochondrial-dependent autoimmunity in membranous nephropathy of IgG4-related disease. EBioMedicine.

[24] Xu J, Zhang W, Xu Y, Shen P, Ren H, Wang W, Li X, Pan X, Chen N (2013). Tacrolimus combined with corticosteroids in idiopathic membranous nephropathy: a randomized, prospective, controlled trial. Contrib Nephrol.

[25] du Buf-Vereijken PW, Feith GW, Hollander D, Gerlag PG, Wirtz JJ, Noordzij TC, Wetzels JF (2004). Restrictive use of immunosuppressive treatment in patients with idiopathic membranous nephropathy: high renal survival in a large patient cohort. QJM.

[26] Cattran DC, Appel GB, Hebert LA, Hunsicker LG, Pohl MA, Hoy WE, Maxwell DR, Kunis CL (2001). Cyclosporine in patients with steroid-resistant membranous nephropathy: a randomized trial. Kidney Int.

[27] Goumenos DS, Katopodis KP, Passadakis P, Vardaki E, Liakopoulos V, Dafnis E, Stefanidis I, Vargemezis V, Vlachojannis JG, Siamopoulos KC (2007). Corticosteroids and ciclosporin a in idiopathic membranous nephropathy: higher remission rates of nephrotic syndrome and less adverse reactions than after traditional treatment with cytotoxic drugs. Am J Nephrol.

[28] Chen M, Li H, Li XY, Lu FM, Ni ZH, Xu FF, Li XW, Chen JH, Wang HY (2010). Tacrolimus combined with corticosteroids in treatment of nephrotic idiopathic membranous nephropathy: a multicenter randomized controlled trial. Am J Med Sci.

[29] Ramachandran R, Hn H, Kumar V, Nada R, Yadav AK, Goyal A, Kumar V, Rathi M, Jha V, Gupta KL, et al. Tacrolimus combined with corticosteroids versus modified Ponticelli regimen in treatment of idiopathic membranous nephropathy: randomized control trial. Nephrology (Carlton). 2016;21:139-46. 10.1111/nep.12569 26205759

